# A Novel Heat Shock Element (HSE) in *Entamoeba histolytica* that Regulates the Transcriptional Activation of the *EhPgp5* Gene in the Presence of Emetine Drug

**DOI:** 10.3389/fcimb.2017.00492

**Published:** 2017-11-29

**Authors:** Alma Nieto, David G. Pérez Ishiwara, Esther Orozco, Virginia Sánchez Monroy, Consuelo Gómez García

**Affiliations:** ^1^Laboratorio de Biomedicina Molecular I, Escuela Nacional de Medicina y Homeopatía, Instituto Politécnico Nacional, Mexico City, Mexico; ^2^Departamento de Infectómica y Patogénesis Molecular, Centro de Investigación y de Estudios Avanzados del Instituto Politécnico Nacional, Mexico City, Mexico

**Keywords:** multidrug resistance, HSE, *Entamoeba histolytica*, *EhPgp5*, emetine, stress

## Abstract

Transcriptional regulation of the multidrug resistance *EhPgp5* gene in *Entamoeba histolytica* is induced by emetine stress. *EhPgp5* overexpression alters the chloride-dependent currents that cause trophozoite swelling, diminishing induced programmed cell death (PCD) susceptibility. In contrast, antisense inhibition of P-glycoprotein (PGP) expression produces synchronous death of trophozoites and the enhancement of the biochemical and morphological characteristics of PCD induced by G418. Transcriptional gene regulation analysis identified a 59 bp region at position −170 to −111 bp promoter as putative emetine response elements (EREs). However, insights into transcription factors controlling *EhPgp5* gene transcription are missing; to fill this knowledge gap, we used deletion studies and transient CAT activity assays. Our findings suggested an activating motif (−151 to −136 bp) that corresponds to a heat shock element (HSE). Gel-shift assays, UV-crosslinking, binding protein purification, and western blotting assays revealed proteins of 94, 66, 62, and 51 kDa binding to the *EhPgp5* HSE that could be heat shock-like transcription factors that regulate the transcriptional activation of the *EhPgp5* gene in the presence of emetine drug.

## Introduction

The multidrug resistance phenotype (MDR) is a phenomenon in which the cell is able to survive in the presence of a drug and shows cross-resistance to a variety of structurally unrelated drugs across membranes in a wide range of organisms (Moons, 2003). The MDR phenotype has been identified in several organisms from mammals to prokaryotes provoking serious problems for the treatment and control of different illness, such as cancer, sepsis, and several parasitoses, such as malaria, leishmaniasis, and others (Juranka et al., 1989).

The P-glycoprotein (PGP) an ATP-dependent membrane-bound transporter is the major protein involved in the MDR phenotype (Mickley et al., 1989). In humans, the PGP is encoded by two *MDR* genes (*MDR1* and *MDR3*) (Chin et al., 1989); in mice, by *mdr1a* and *mdr1b* (Cui et al., 2009); in *Leishmania major*, by *lmmdr1* and *lmmdr2;* and in *Plasmodium falciparum*, by *pfmdr1* and the *pfmdr2* (Grogl et al., 1991). Interestingly, the largest *mdr* gene family described until now is present in *Entamoeba histolytica* (Orozco et al., 2001), the protozoan parasite responsible for human amoebiasis, which causes an estimated 50 million cases of invasive disease and 70,000 deaths per year (World Health Organization, 1998). The multigenic amoeba *mdr* family is composed of *EhPgp1, EhPgp2, EhPgp5*, and *EhPgp6* genes. A differential gene expression pattern has been documented in drug-sensitive (clone A) and drug-resistant (clone C2) trophozoites (Descoteaux et al., 1995). *EhPgp1* is constitutively expressed in trophozoites from clones A and C2 (Gómez et al., 1996). In contrast, the *EhPgp5* gene showed inducible expression due to the presence of emetine in culture medium (Descoteaux et al., 1995). The transcript of this gene was not detected in clones A and C2 growth without drug. However, when C2 trophozoites were cultured in the presence of emetine, expression of the *EhPgp5* gene increased in a drug concentration-dependent manner (Descoteaux et al., 1995). Although an increase in the *EhPgp5* mRNA half-life was reported (López-Camarillo et al., 2003), its expression was mainly dependent on transcriptional activation (Pérez et al., 1998). The *EhPgp5* promoter region, from −170 to +30 bp, was able to efficiently drive the expression of CAT reporter gene when trophozoites from clones A and C2 were cultured under drug pressure (Pérez et al., 1998), suggesting that specific emetine response elements (EREs) were present in this region. Additionally, functional assays suggested that the putative ERE could be localized between −170 and −111 bp of the *EhPgp5* gene promoter (Nieto et al., 2005).

To investigate the relevance of ERE to *EhPgp5* gene transcription, deletions, and transient CAT activity studies were performed here. Our findings suggested an activating motif (−151 to −136 bp) that corresponds to a heat shock element (HSE). Gel-shift assays, UV-crosslinking, binding protein purification, and western blotting assays revealed proteins binding to the *EhPgp5* HSE that could be heat shock-like transcription factors, which regulate transcriptional activation of the *EhPgp5* gene in the presence of emetine drug.

## Materials and methods

### *E. histolytica* cultures

Trophozoites of clones A and C2 (strain HM1: IMSS) (Orozco et al., 1985) were axenically cultured in TY1-S-33 medium (Diamond et al., 1978). Emetine stress was exerted by the incubation of trophozoites from clones A or C2 in 8 or 20 μM emetine, respectively, for 24 h.

### *In Silico* search for potential transcription binding sites

The TRANSFACT database (MatInspector software) was used to identify potential transcription factor binding sites in the *EhPgp5* promoter (Matys et al., 2006).

### Plasmid constructions

**Figure 1:**
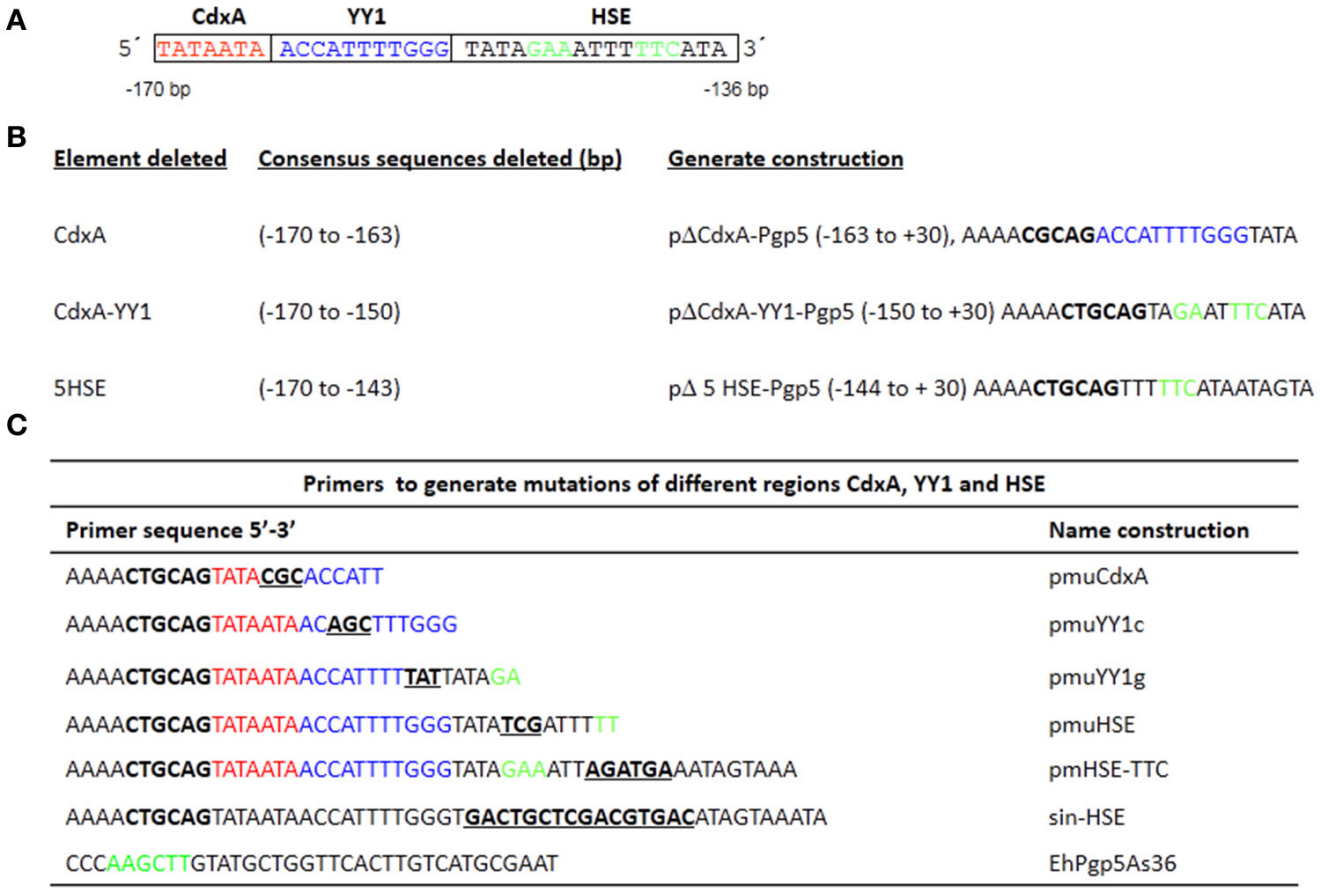
Plasmid constructions in the *EhPgp5* gene core promoter. **(A)** Schematic representation of three consensus sequences in the *EhPgp5* gene promoter (−170 to −136 bp). CdxA, YY1, and HSE motifs are marked in red, blue, and green in the rectangles, respectively. **(B)** Constructs generated by deletion of each consensus sequence of the *EhPgp5* gene core promoter (−170 to −136 bp). **(C)** Primers used to generate different mutations in CdxA, YY1, and HSE motifs. Mutations are underlined and bolded in black. Restriction sites for *Pst*I and *Hind* III are bolded in black. The last sequence is the antisense primer used for all constructs.

To perform transfection experiments, several plasmids were constructed by inserting PCR-amplified *EhPgp5* gene core promoter fragments into multiple cloning sites of the pBSCAT-ACT plasmid (Gómez et al., 1998). The constructs performed are shown in Figures 1A,B. Constructs with specific mutations in the CdxA, YY1, and HSE regions were performed by PCR amplification using the primers shown in Figure 1C. Promoter-less (pBSCAT-ACT) and minimal p259Pgp5 promoter constructs (Pérez et al., 1998) were used as negative and positive controls, respectively. The pA5′A3′CAT plasmid (Nickel and Tannich, 1994), which contains a 480 bp fragment from the *actin* gene promoter, was used as an internal control of transfection efficiency. Correct sequence orientation of the cloned fragments was determined by sequencing analysis.

### Transfection and cat assays

Transfection assays were performed by electroporation (Nickel and Tannich, 1994). Briefly, 10^6^ trophozoites of clones A and C2 were transfected with 100 μg of the different plasmid constructs (ΔCdxA-Pgp5, ΔCdxA-YY1-Pgp5, Δ5HSE-Pgp5, pmuCdxAPgp5, pmuYY1cPgp5, pmuYY1gPgp5, pmuHSEPgp5, pmHSE-TTCPgp5, p-sinHSEPgp5, p259Pgp5, pBSCAT-ACT, and pA5′A3′CAT). Electroporated trophozoites were transferred into plastic flasks (Nalgene, NY, USA) containing 50 ml of TYI-S-33 medium and were incubated for 48 h at 37°C. Two hours after electroporation, we added 8 μM emetine to clone A or 20 μM emetine to clone C2. CAT activities were measured by two-phase diffusion assay using 5 μg of trophozoite extracts and 200 μl of chloramphenicol (1.25 mM), which were incubated with [^14^C]-butyril-CoA (NEN Life Science Products, MA, USA) for 2 h. Protein concentrations were determined using Bradford's methods (Bradford, 1976). CAT activities were expressed as the cpm of the butyrylated derivatives. The background obtained from the trophozoites transfected with the pBSCAT-ACT plasmid was subtracted from the measurements given by the plasmids containing the different promoter constructions. CAT activities were determined in the linear range of assays, representing three independent experiments performed in triplicate. The efficiency of the transfection experiments was monitored by the activity of pA5′A3′CAT plasmid (Nickel and Tannich, 1994).

### Nuclear extracts (NEs)

NEs were prepared from trophozoites of clones A and C2 grown in the presence [clones A_(8)_ and C2_(20)_] or absence (clones A and C2) of emetine, following Schreiber et al.'s protocol (Schreiber et al., 1989), modified by Gómez et al. (1998). Protein concentrations were determined by Bradford's methods (Bradford, 1976).

### Electrophoretic mobility gel-shift assays

Double-stranded oligonucleotides of the *EhPgp5* HSE sequence from −150 to −137 bp (HSE-S-16, 5′-ATAGAAATTTTTCATA-3′) were synthesized and labeled using T4 polynucleotide kinase (Invitrogen, CA, USA) in the presence of [γ-^32^ P]-ATP. Specific activity was determined by scintillation counting. Gel-shift assays were performed in triplicate as described previously (Gómez et al., 1998). Briefly, we used 0.5–1 ng of labeled probes (10,000 cpm), 1 μg of poly [d (I-C)] (Amersham Pharmacia Biotech, NJ, USA), 20 μg of NE from trophozoites and 10% glycerol in DNA–protein binding buffer. For competition assays, 150-fold molar excess of unlabeled oligonucleotides was incubated with NE at 4°C for 10 min prior to adding the radiolabeled probes. As competitors, we used poly [d (I-C)] and the double-stranded oligonucleotides S-34 (5′-TATAATAACCATTTTGGGTATAGAAATTTTTCAT-3′) and HSEm (5′-ATATCGATTTTTCATA-3′).

### UV-crosslinking and western blot experiments

UV-crosslinking assays were performed according to Ausubel et al. (1994) with some modifications. Standard gel-shift mixtures using NE from trophozoites of both clones grown in the absence or presence of emetine and the radiolabeled HSE probe were scaled up five-fold and were exposed to irradiation in a 312 nm UV-transilluminator (Bio-Rad, CA, USA) for 10 min at 4°C; then, the proteins were separated by 15 % SDS-PAGE (Laemmli, 1970). The gels were dried and analyzed by autoradiography. DNA cross-linked proteins were analyzed by western blot using the standard procedure (Ausubel et al., 1994). The membranes were incubated with 2 μg of human anti-HSF1 (H-311, Santa Cruz Biotechnology, TX, USA) in 5% non-fat dry milk and 0.05% Tween-20 in phosphate-buffered saline (PBS), with a pH of 7.4, overnight at 4°C. The proteins were revealed by peroxidase-conjugated anti-rabbit secondary antibodies (ZYMED Laboratories, CA, USA) (1:3000) and were immunodetected by a chemiluminescence system (ECL Plus ™, Amersham Pharmacia Biotech, NJ, USA). The assays were repeated at least three times for each studied condition.

### Purification of *E. histolytica* proteins

*E. histolytica* proteins that bind to the HSE sites of the *EhPgp5* gene core promoter were partially purified three separate times under non-denaturing conditions using a DNA-binding protein purification kit (Roche, CA, USA) and NE from trophozoites of both clones A_[8]_ and C2_[20]_. Concatemeric polynucleotides were prepared by PCR amplification of the HSE probe. The oligomer was coupled to magnetic particles coated with streptavidin, as described by the manufacturer. Seventy-five micrograms of NE from trophozoites were mixed with the magnetic particles in protein buffer, poly [d (I-C)] and poly-L-lysine. Then, NE was incubated at 4°C for 60 min. After extensive washing, proteins bound to the particles were eluted with elution buffer containing 2 M KCl. All of the fractions were analyzed by 10% SDS-PAGE (Laemmli, 1970). The gels were silver stained or transferred to nitrocellulose membranes for western blotting assays. Purified fractions were added to DNA–protein binding reactions to perform EMSA, as described above.

### Statistical analysis

All of the data are expressed as the means ± SDs. IBM SPSS software, version 23, was used to compare all of the groups to each other. For all tests, *p* < 0.05 was considered significant.

## Results

### Structural characterization of the −170 to −111 bp of the *EhPgp5* gene core promoter

Previously, a functional region of 59 bp (−170 to −111 bp) of the *EhPgp5* gene promoter was involved in the induction of *EhPgp5* gene expression (Nieto et al., 2005). To detect specific transcription elements within this region, we performed a bioinformatic search for the presence of consensus sequences of different transcription factors within the 59-bp *EhPgp5* gene promoter region, using the TransFact database. The structural analysis of this sequence revealed the presence of putative consensus sequences for CdxA (−170 to −164 bp) and YY1 (−163 to −153 bp) transcription factors and, interestingly, a HSE at −151 to −136 bp (Figure 1A).

### Functional identification of the ERE in the region from −170 to −111 bp of the *EhPgp5* gene core promoter

To identify whether these cis-regulatory elements direct specific *EhPgp5* gene expression in *E. histolytica* drug-sensitive [A_(8)_] and drug-resistant [C2_(20)_] clones grown in the presence of 8 and 20 μM emetine, respectively, we performed a series of mutations and deletions within the 59 bp region. Different constructs were generated, as described in the Materials and Methods section (Figures 1B,C). These plasmids contain internal deletions of CdxA, YY1, or HSE sequences.

**Figure 2:**
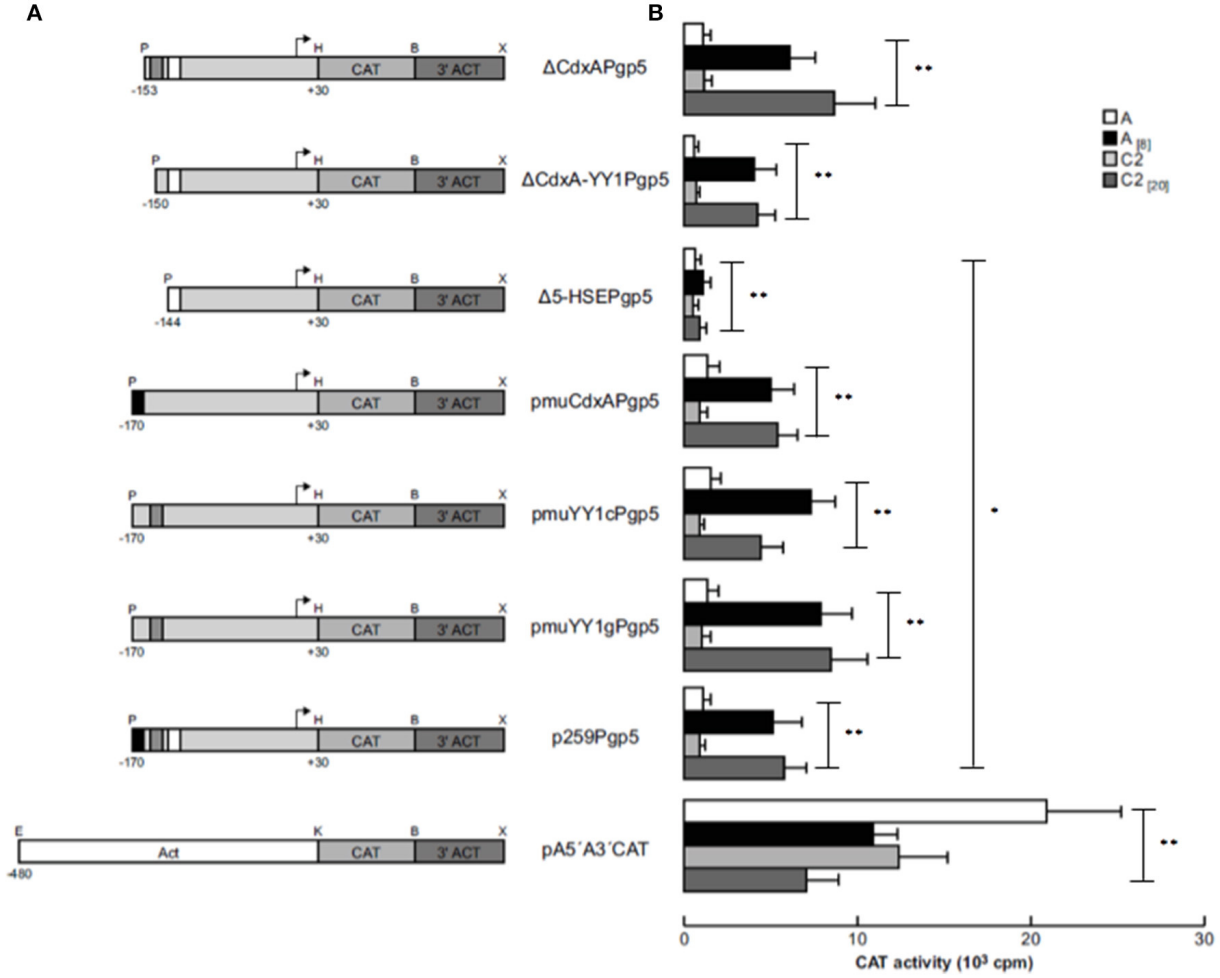
Functional identification of the ERE in the region from −170 to −111 bp of the *EhPgp5* gene core promoter. **(A)** Schematic representation of the constructs used for the transfection assays. All of the plasmids contain the CAT reporter gene and the 3′-flanking actin region (3′ ACT). Arrows, transcription initiation sites. P, *Pst*I; H, *Hind*III; B, *Bam*HI; X, *Xho*I; E, *EcoR*I; K, *Kpn*I. CdxA, YY1, and HSE motifs are in black, gray and white boxes, respectively. **(B)** Bars show the average of CAT activity (cpm) of the transfected plasmids obtained by the two-phase diffusion assays ± SD, representative of three independent experiments performed in duplicate (^*^p < 0.05; ^**^p < 0.001). The background given by the trophozoites transfected with pBSCAT-ACT plasmid was subtracted in all of the experiments. The efficiency of the transfection assays was monitored by the activities given by the pA5′A3′CAT plasmid.

Transfection assays of all constructs were performed in trophozoites from clones A and C2. In transfected trophozoites of both clones grown without emetine, no CAT activities were detected with any constructs, compared to the CAT activity developed by pA5′A3′CAT plasmid (Figure 2). However, when emetine was added to the culture medium after electroporation, we observed significantly higher CAT activities with all the constructs, similar to the activity shown by the *EhPgp5* gene core promoter (p259Pgp5 plasmid) (Figure 2), except with the pΔ5-HSEPgp5 plasmid. No significant differences were observed in the CAT activities shown by ΔCdxAPgp5, ΔCdxA-YY1Pgp5, pmuCdxAPgp5, pmuYY1cPgp5, and pmuYY1gPgp5, suggesting that CdxA and YY1 putative consensus sequences were not involved in *EhPgp5* gene transcriptional activation. In contrast, the construct pΔ5-HSEPgp5 that does not contain six of 16 bases that form the putative HSE element (ataGAAatttTTCata) displayed significant CAT activity reductions of 80 and 78% in trophozoites from clones A_[8]_ and C2_[20]_, respectively.

### The HSE element is indispensable to activating *EhPgp5* gene transcription

**Figure 3:**
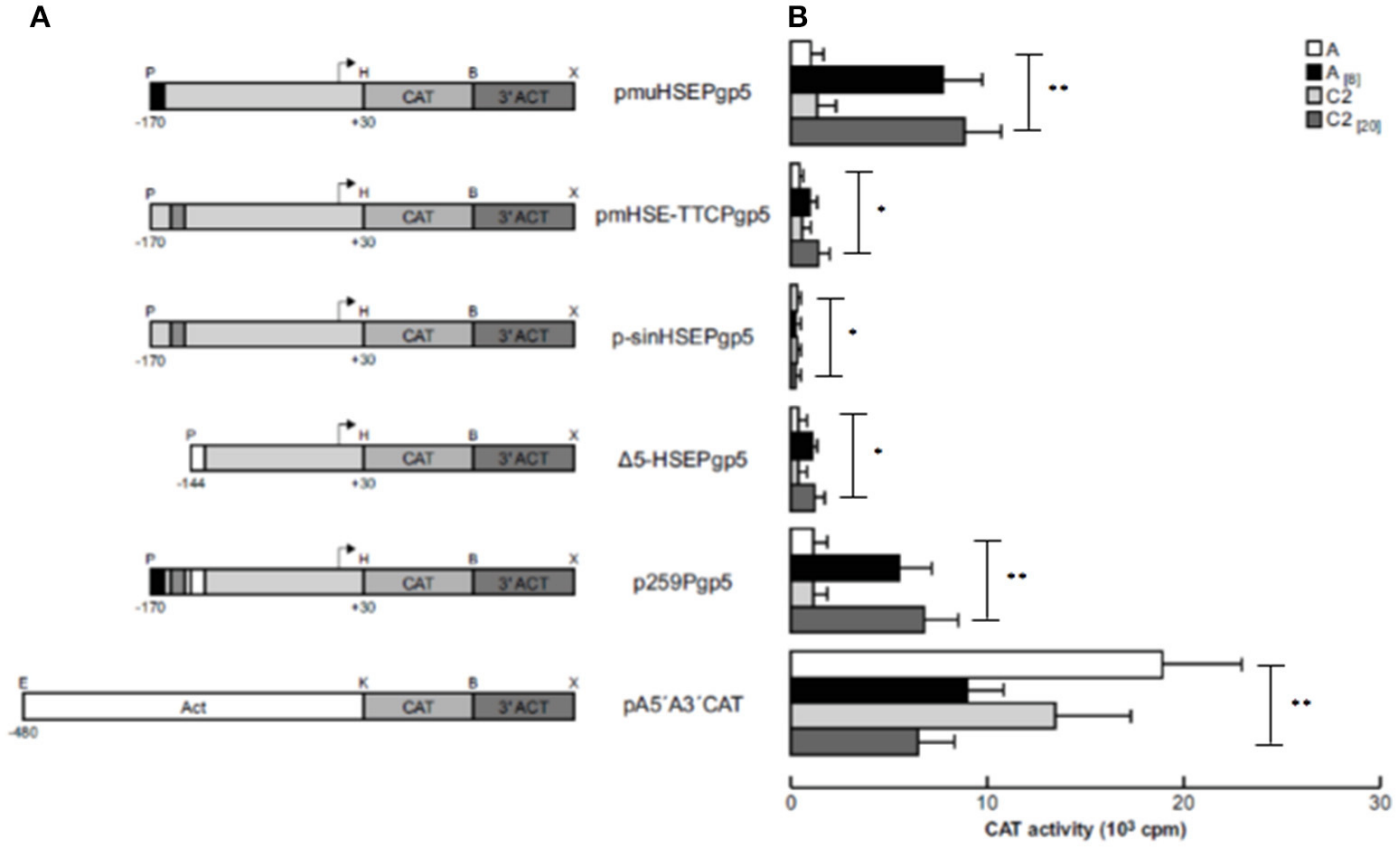
Functional identification of the putative HSE element in the region from −170 to −111 bp of the *EhPgp5* gene core promoter. **(A)** Schematic representation of the constructs used for the transfection assays. All of the plasmids contain the CAT reporter gene and the 3′-flanking actin region (3′ ACT). Arrows, transcription initiation sites. P, *Pst*I; H, *Hind*III; B, *Bam*HI; X, *Xho*I; E, *EcoR*I; K, *Kpn*I. CdxA, YY1, and HSE motifs are in black, gray and white boxes, respectively. **(B)** Bars show the average CAT activity (cpm) of the transfected plasmids obtained by the two-phase diffusion assays ± SD, representative of three independent experiments performed in duplicate. The background given by the trophozoites transfected with pBSCAT-ACT plasmid was subtracted in all of the experiments. The efficiency of transfection assays was monitored by the activities given by the pA5′A3′CAT plasmid. Vertical bars and asterisks indicate significant differences (^*^p < 0.05; ^**^p < 0.001).

To demonstrate that the putative HSE (−151 to −136 bp) is responsible for activating *EhPgp5* gene expression by emetine, we generated three new constructs introducing point mutations into the putative HSE. pmuHSEPgp5 contains three nucleotide replacements in the central inverted repeated GAA of HSE (ATA**GAA**ATTT**TTC**ATA → ATA**TCG**ATTTTTCATA); pmHSE-TTCPgp5 had three base changes in the inverted repeated TTC and its flanking bases (ATA**GAA**ATTT**TTC**ATA → ATAGAAATTA**GAT**GAA), and in p-sinHSEPgp5, the HSE sequence was completely replaced (GACTGCTCGACGTGAC). We transfected these constructs into trophozoites from clones A and C2 and then cultured them in the absence or presence of 8 or 20 μM emetine, respectively. The results showed that all of the constructs presented CAT activities in trophozoites from both clone cultures without emetine (Figure 3). However, a dramatic difference in expression was observed when emetine was added to the cultures, and the plasmid pmuHSEPgp5 presented higher CAT activity, similar to that shown by the p259Pgp5 minimal promoter (Figure 3). The activities observed were similar in both clones [A_(8)_ and C2_(20)_], suggesting that the changes introduced into the GAA sequence from the HSE did not affect the transcriptional activation. Interestingly, the plasmid pmHSE-TTCPgp5 presented a strong CAT activity reduction in trophozoites from both clones, suggesting that the second repeat of the HSE was indispensable to *EhPgp5* gene expression. Moreover, when the HSE was eliminated, CAT reporter gene activity was not detected, either in trophozoites of clone A_[8]_ or in trophozoites of clone C2_[20]_.

### The *EhPgp5* HSE is recognized by nuclear proteins from *E. histolytica* trophozoites from sensitive and resistant clones

**Figure 4:**
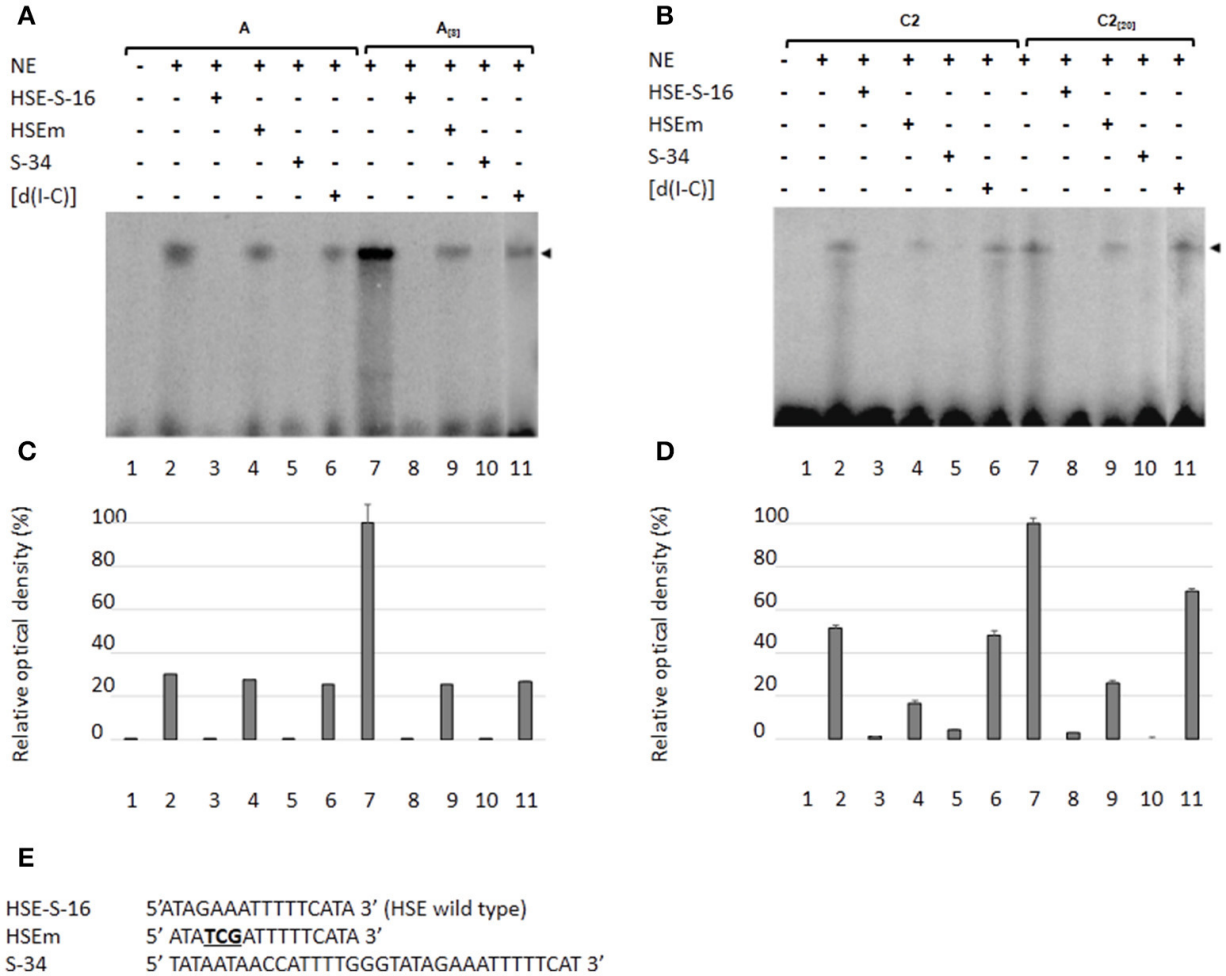
Nuclear protein binding with the putative HSE element of the *EhPgp5* gene core promoter. Gel shift assays were performed with 20 μg of NE from clones: **(A)** A and A_[8]_, **(B)** C2 and C2_[20]_, 1 ng of [γ-^32^P]-radiolabeled double-stranded fragment of different unlabeled oligonucleotides (150-fold molar excess). Lane 1, free probe; lanes 2 and 7, no competitor; lanes 3 and 8 specific competitor unlabeled straight (150-fold molar excess); lanes 4 and 9, specific competitor with the HSE mutated at the repeated GAA; lanes 5 and 10, specific competitor containing the CdxA, YY1, and HSE consensus sequences; lanes 6 and 11 unspecific competitor (350-fold molar excess of poly [d(I–C)]). Arrowheads indicate the specific complexes formed. **(C,D)** Densitometric analysis of DNA-binding interaction with NE from clones A and A_[8]_ and C2 and C2_[20]_, respectively. **(E)** Sequence of the *EhPgp5* HSE used as a probe (HSE-S-16) and the competitors (HSEm and S-34). Mutations are underlined and bolded in black. Data presented in this figure are representative of three independent experiments.

As a second approach to gaining insight into the role of HSEs in the promoter activity of the *EhPgp5* gene, we analyzed DNA–protein interactions using gel shift and competition assays. Gel electrophoretic mobility shift assays were performed to obtain evidence of nuclear protein binding to the putative HSE. Double-stranded synthetic oligonucleotides containing the *EhPgp5* HSE were incubated with NE from clones A, A_[8]_, C2, and C2_[20]_. Additionally, oligonucleotides containing the wild-type consensus HSE and the HSEm mutated at the repeated GAA and the region of 34 bp of the *EhPgp5* promoter from −170 to −136 bp, which contains the CdxA, YY1, and HSE sequences, were used as specific competitors. As demonstrated in Figures 4A,B, the HSE was specifically recognized by nuclear proteins from *E. histolytica* clones grown in the presence or absence of emetine, forming one DNA–protein complex that had identical electrophoretic mobility using NE from both clones. Interestingly, the intensity of the complex was enhanced when the DNA probe was incubated with NE from the trophozoites from clones A_[8]_ and C2_[20]_ (Figures 4A–D, lanes 2 and 7). Competition assays showed that formation of the complex with the HSE probe was efficiently blocked by the wild-type HSE and the S34 oligonucleotides when a 150-fold molar excess was used (Figures 4A–E, lanes 3, 5, 8, and 10) but not with the HSEm, for which the DNA–protein complexes only diminished (Figures 4A–E, lanes 4 and 9), suggesting that the DNA–protein interaction was partially affected by changing the GAA repeated sequence. However, this change did not interfere with the *EhPgp5* promoter activity (Figures 4A,B). In contrast, when poly [d(I-C)] was used as a non-specific competitor (350-fold molar excess), the DNA–protein complex formation was not affected (Figures 4A–D, lanes 6 and 11).

### Molecular weights of the nuclear proteins binding to the *EhPgp5* HSE

**Figure 5:**
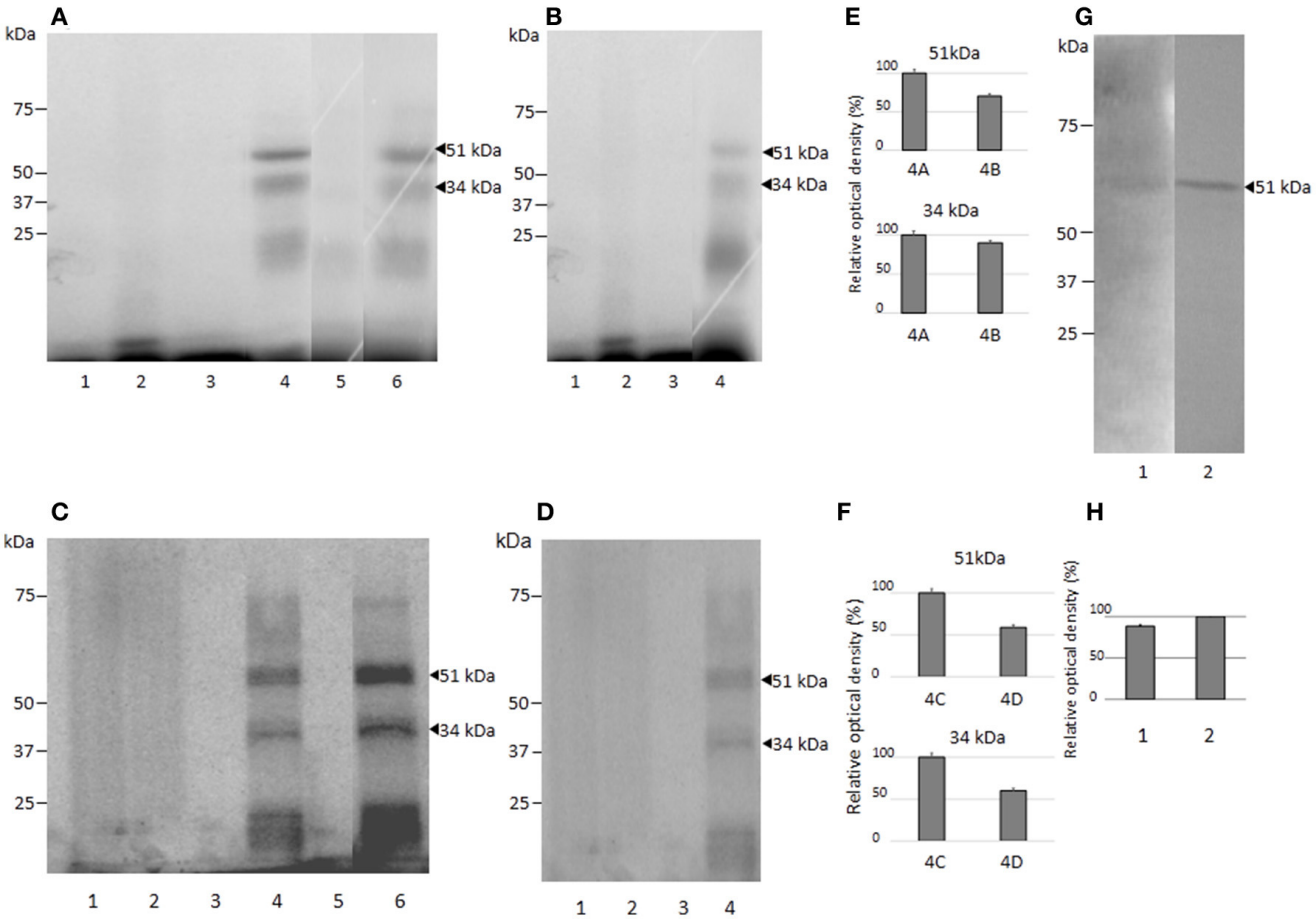
Molecular weight of the nuclear proteins binding to the *EhPgp5* HSE and their immunorelation to human HSF. UV-crosslinking analyses after EMSA in A and C2 clone grown in the presence **(A,C)** or in the absence **(B,D)** of emetine. One hundred micrograms of NE from clones A **(A,B)** and C2 **(C,D)** trophozoites were UV cross-linked to 5 ng of the γ^32^P-ATP radiolabeled HSE probe for 10 min at 4°C, and DNA–protein complexes were electrophoresed through 15% SDS-PAGE, followed by exposure to X-ray film. Lane 1, UV untreated HSE probe; lane 2, UV irradiation of HSE probe; lane 3, UV untreated complete binding reaction; lane 4, UV treatment of complete binding reaction; lane 5, probe (150-fold molar excess); lane 6, unspecific competitor poly [d(I-C)]. Arrowheads, DNA–protein complexes after correction for the bound probe. Relative densitometry of each protein in clones A_[8]_ and A **(E)** and C2_[20]_ and C2 **(F)**. **(G)** Western blot assay of UV cross-linked DNA–protein complexes of clones A_[8]_ and C2_[20]_ using heterologous antibodies to anti-HSF1 from humans with their corresponding densitometric values. **(H)** Lane 1, clone A_[8]_; and lane 2, clone C2_[20]_. All assays were performed on three different occasions.

To identify the nuclear proteins binding to the *EhPgp5* HSE, we performed UV-crosslinking assays after gel shift. Two main proteins of 51 and 34 kDa after correction for the bound probe (61 and 44 kDa) were found in both clones incubated with or without emetine (Figure 5). The HSE-protein interactions were specific due to complex formation being prevented by a 150-fold molar excess of cold oligonucleotide (lane 5), while binding proteins were not inhibited using the unspecific competitor poly [d (I-C)] (lane 6), indicating the specificity of the complexes. The bands obtained using the NE trophozoites from both clones grown in the presence of emetine (Figures 5A,C,E,F), were more intense than the bands obtained using the NE trophozoites from both clones grown in the absence of emetine (Figures 5B,D–F).

### Putative heat shock transcription factor binds to the *EhPgp5* HSE

To identify the proteins interacting with the *EhPgp5* HSE, we searched for putative heat shock transcription factors (HSTF) by western blot assays of the UV-crosslinked complexes from trophozoites of clones A, A_[8]_, C2, and C2_[20]_ using the human heterologous antibody anti-HSF1. As shown in Figure 5G, a band of 51 kDa was detected after correction for the bound probe from the DNA–protein complex formed with the HSE element. The band was stronger (20%) in C2_[20]_, trophozoites (Figure 5H). In trophozoites from both clones grown without emetine, a very slight signal was detected (data not show).

### Partial purification of the proteins binding to the *EhPgp5* HSE

**Figure 6:**
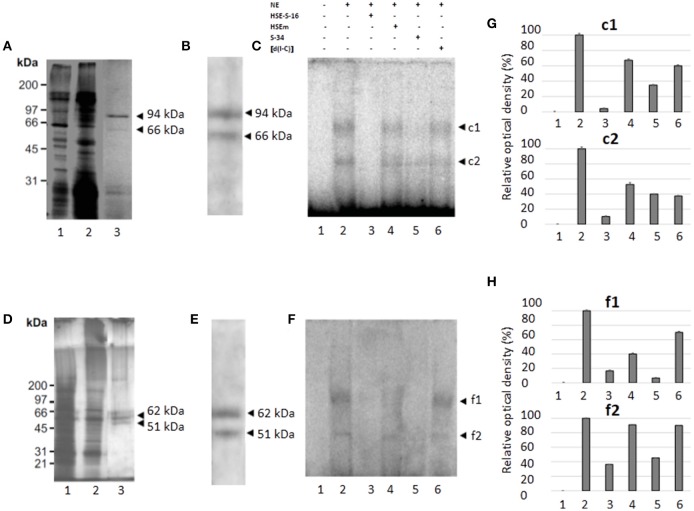
Purification, immunodetection, and DNA-interaction of the nuclear proteins that bind to the HSE element **(A,D)**. 10% SDS-PAGE of protein fractions obtained from the purification protocol of NE from clones A_[8]_ and C2_[20]_, respectively. Lane 1, NE (5 μg); lane 2, non-retained fraction; and lane 3, partially purified fraction. **(B,E)** Western blot analysis of fractions obtained from the purification protocol of NE of clones A_[8]_ and C2_[20]_, respectively. Arrowheads, silver stained polypeptides detected by antibodies against HSF1 from humans. **(C,F)** EMSA using [γ-^32^P]-ATP radiolabeled HSE probe and partially purified fraction from clones A_[8]_ and C2_[20]_, respectively. Lane 1, free probe; lane 2, no competitor; lane 3, specific competitor unlabeled straight (150-fold molar excess); lane 4, specific competitor with the HSE mutated; lane 5, specific competitor containing the CdxA, YY1, and HSE consensus sequences; lane 6, unspecific competitor (350-fold molar excess of poly[d(I–C)]). Arrowheads, specific complexes formed. **(G,H)** Relative amounts of DNA binding protein complexes with partial purification proteins of clones A[8] and C2[20], respectively. Each bar indicates the mean ± SE. Data presented in this figure are representative of three independent experiments.

We performed partial purification of proteins that bind to the HSE using the DNA-binding protein purification kit from Roche Molecular Biochemical (Roche, CA, USA), NE from trophozoites of clones A_[8]_ and C2_[20]_ and a concatamerized oligonucleotide containing the HSE. Two main and defined enrichment purified proteins of ~94 and 66 kDa, respectively, were detected on a silver-stained SDS-polyacrylamide gel in NE from trophozoites of clone A_[8]_, (Figure 6A, lane 3), while two proteins of 62 and 51 kDa were purified from the NE from the trophozoites of clone C2_[20]_ (Figure 6D, lane 3). To determine whether purified proteins were also detected by the HSTF1 heterologous antibody, we performed western blot assays. The results showed that both pairs of proteins, 94 and 66 kDa and 62 and 51 kDa from A_[8]_ and C2_[20]_ trophozoites, respectively, were recognized by the human anti-HSTF1 (Figures 6B,E). These results supported the idea that the proteins that binding to the *EhPgp5* HSE could be putative heat shock like transcription factors.

Furthermore, the semipurified proteins from both clones were used to determine whether these proteins could bind to the HSE sequence. As shown in Figures 6C,F,G,H (lane 2), two specific DNA–protein complexes were observed with the semipurified proteins of clones A_[8]_ and C2_[20]._ The complexes were highly specific due to being completely inhibited by a 150-fold molar excess of the same unlabeled wild-type HSE and S34 oligonucleotides (lanes 3 and 5), and 350-fold molar excess of poly [d(I-C)] did not affect complex formation (lane 6). Interestingly, the HSEm did not affect complex formation (Figures 6C,F–H lane 4).

## Discussion

The *EhPgp5* gene and its encoded P-glycoprotein (EhPGP5) have functional relevance in different mechanisms of *E. histolytica* parasites. On the one hand, the antisense inhibition of gene expression enhances the PCD of trophozoites in the presence of G418; on the other hand, gene overexpression alters the chloride-dependent currents and confers drug resistance to emetine (Pérez et al., 1998; Bañuelos et al., 2002; Delgadillo et al., 2002; Medel Flores et al., 2013).

Therefore, due to the relevance of EhPGP5 for different cellular processes, its transcription must be finely regulated. To determine the molecular effectors up-regulating *EhPgp5* gene expression, our group performed the structural and functional characterization of *EhPgp5* gene promoter (Pérez et al., 1998) in trophozoites growth in the absence and presence of emetine, delimiting a 59 bp region at the position from −170 to −111 bp, in which putative EREs were found (Nieto et al., 2005).

Here, we investigated for the first time the functional roles of motifs found in promoter activity. Consensus DNA-binding sequences for different transcription factors are well conserved throughout evolution, and different databases of transcription factors and promoters have been established to develop algorithms, as well as computer methods to predict sequence target sites for specific DNA-binding proteins (Boeva, 2016). The bioinformatic screening revealed three consensus sequences for CdxA, YY1, and HSE, recognized by the CdxA, Ying Yang 1 (YY1), and HSTF proteins, respectively (Perisic et al., 1989; Shi et al., 1991; Margalit et al., 1993).

In *E. histolytica*, seven putative HSEs located in *EhrabB*, three in *Ehhsp100* and four in *Ehmlbp* gene promoter have been reported (Bernes et al., 2005; Romero-Díaz et al., 2007; Katz et al., 2012). Functional CAT assays driven by *EhrabB* promoter demonstrated that CAT activity increased by two times when trophozoites were exposed to heat shock stress (42°C) (Romero-Díaz et al., 2007). Deletion of HSEs from *Ehmlbp* gene promoter down-regulated CAT reporter gene expression in trophozoites exposed to heat shock (Katz et al., 2012), while the activity of the HSEs present in *Ehhsp100* gene promoter has not yet been studied. Comparison of the HSE from the *EhPgp5* gene promoter with those reported in *E. histolytica* showed greater similarity with the HSEs of the *Ehhsp100* gene promoter. Both sequences were formed by two motifs; however, the orientation of the *EhPgp5* HSE (ATA**GAA**ATTT**TTC**ATA) is head-tail (GAA and TTC), while that of the *Ehhsp100* is head-head (AAG**GAA**CTT**GAA**GAA), containing a gap of four and three bases between them, respectively (Bernes et al., 2005). In contrast, Tchénio et al. (2006) that osteosarcoma cells became doxorubicin resistant by overexpression of the MDR1 gene, induced by the HSE. These results together supported the idea that HSE within the *EhPgp5* gene promoter is relevant for its expression in trophozoite culture under emetine drug pressure, which is a stress situation. Functional analyses of the HSE (nGAAnnTTCn) in other organisms have demonstrated that the GAA and TTC motifs contain indispensable bases for DNA–protein interactions and consequently for promoter activation (Enoki and Sakurai, 2011). Mutations or deletions of these motifs decreased or abolished the promoter activity of the *RPN4* and *PDR3* genes in *Saccharomyces cerevisiae* and *BnGolS1* in plant cells in a similar manner to that we observed for the *EhPgp5* HSE (Hahn et al., 2006; Lang et al., 2017). We demonstrated here that CAT activity was abolished by complete HSE substitution or by TTC (GAT) mutation, strongly supporting that emetine increases the activity of the *EhPgp5* gene promoter via HSE.

Using DNA–protein interactions, western blots from crosslinked and semipurified proteins employing heterologous HSTF antibodies indicated that HSE interacts with an HSTF-like factor present in both sensitive and resistant trophozoites; however, functional CAT assays demonstrated that the promoter was active only in the presence of emetine, suggesting that binding of HSTF-like factor is not sufficient to drive promoter activity. Several pieces of evidence from other systems have shown that HSTF factors present a multistep activation mechanism (trimerization, phosphorylation, and translocation to nucleus) to activate gene expression (Sorger and Pelham, 1988; Høj and Jakobsen, 1994; Sandqvist et al., 2009). These factors are activated in the presence of stress induced by heat, oxidative stress, heavy metals, bacterial toxins, and drugs between others (Høj and Jakobsen, 1994; Akerfelt et al., 2007). In amoebas, it was reported that the HSE from *Ehhsp100* promoter was recognized by a 37 kDa protein when the trophozoites were exposed to heat shock (Bernes et al., 2005), while a 25 kDa protein bound to the HSE in *Ehmlbp* promoter also under heat shock (Katz et al., 2012).

Different experimental procedures performed here showed that HSE interacts with 94, 66, and 51 kDa proteins in sensitive trophozoites, while in resistant ones, HSE interacts with 62 and 51 kDa, both recognized by HSTF antibodies.

Differences found in the molecular weights of EhHSTF-like proteins interacting with the HSE in sensitive and resistant clones could be due to: (i) translational modifications; (ii) formation of homodimers or homotrimers; or (iii) interactions with other proteins, for example, molecular weight changes in human HSFs by different posttranslational modifications, mainly phosphorylation (Sorger et al., 1987; Larson et al., 1988; Baler et al., 1993; Sarge et al., 1993). These factors are able to trimerize or interact with different proteins, such as CHIP (C-terminus of HSP70-interacting protein) (Murata et al., 2003), DDL-1 and DDL-2, forming a complex that stabilizes HSF1 monomers (Chiang et al., 2012), or with HSP70 and HSP90 to keep HSTF1 in an inactive state preventing trimerization (Zou et al., 1998) or even more perhaps interacting with other human HSFs (HSF1, HSF2, HSF3, and HSF4) (Rabdiran et al., 1991; Schuetz et al., 1991; Nakai et al., 1997). In this parasite, eight Hsps (101, 100, 90, 70, 60, 40, 20, and 10) have been identified, and some of them have been characterized, showing their participation in heat shock stress (Bernes et al., 2005; Ximénez et al., 2017). These results suggest that amoebas might contain an HSTF demonstrated in other organisms, which is responsible for controlling the expression of the *hsp* genes (Driedonks et al., 2015). In the literature, it has been described that HSF is a master regulator of stress-responsive genes among organisms as distantly related as bacteria and humans (Guertin et al., 2010). Moreover, in the *E. histolytica* genome, seven putative HSTFs are present that could interact with the HSE of the *EhPgp5* gene promoter in trophozoites grown in the presence of emetine (Bernes et al., 2005; Loftus et al., 2005; Macías-Arguelles, in preparation). However, we do not know which of the seven factors is being expressed and is regulating *EhPgp5* gene expression. HSFs are characterized by their ability to bind to the HSE; but, in organisms containing different HSFs, such as humans, mice, and chickens, it has been described that each is able to regulate different genes (Driedonks et al., 2015).

The findings presented here provide evidence of the participation of a novel HSE that requires a putative HSTF-like factor(s) to regulate the transcriptional activation of the multidrug-resistant *EhPgp5* gene in trophozoites exposed to emetine, as has been reported in human MDR1 multidrug-resistance gene. However, the underlying mechanisms explaining how a putative EhHSTF recognizes and binds to the HSE, how emetine activates the *EhPgp5* promoter via EhHSTF, and which of the seven EhHSTFs binds to the HSE remain to be discovered.

## Author contributions

AN: Data acquisition and analysis or for the work. DP: interpretation of data and revision of intellectual content. EO: Revision of intellectual content. VS: Draft the work and revision of intellectual content. CG: Design of the work and revision of intellectual content.

### Conflict of interest statement

The authors declare that the research was conducted in the absence of any commercial or financial relationships that could be construed as a potential conflict of interest. The handling Editor declared a shared affiliation, though no other collaboration, with all the authors.
